# Developing and Validation of Identifying People in Risk of Addiction Questionnaire (I.P.R.A)

**DOI:** 10.5812/ijhrba.8101

**Published:** 2013-03-12

**Authors:** Jafar Anisi, Mohammad Hossein Bahadori, Marziyeh Jahanbakhsh

**Affiliations:** 1 Behavioral Sciences Research Center, Baqiyatallah University of medical sciences, Tehran, IR Iran

**Keywords:** Drug Addiction, Questionnaires, Validation Studies

## Abstract

**Background:**

Drug addiction is considered as a problem of the new century which has destructive consequences for both family and society. This ominous phenomenon resulted from many factors. Present research aimed at recognition of inter-personal factors related to addiction and were conducted through a questionnaire to identify the youths at risk of addiction.

**Objectives:**

Present research aimed at recognition of inter-personal factors related to addiction and were conducted through a questionnaire to identify the youths at risk of addiction.

**Materials and Methods:**

The design of the present research is correlational analytic. The population consists of all young addicted or non-addicted people between the ages 18 to 35 and the sample consists of 82 addicted and 223 non-addicted young people in Tehran who were selected randomly and simply. The initial form included 120 questions which were administered on the sample in three stages. The data was analyzed through descriptive statistic and factor analysis.

**Results:**

In this questionnaire four factors of depression and miserableness, having a positive attitude to drug, stress and anxiety and finally seeking high levels of excitement were respectively the strongest factors in predicting the risk of drug-abuse and addiction. The validity of the questionnaire which consists of 75 questions in the final form was calculated through internal consistency. Cronbach alpha of the whole questionnaire was 0.97, which that of the factor of depression and miserableness was 0.96, the factor of a positive attitude to drug was 0.93, the factor of stress and anxiety was 0.90 and the factor of high excitement-seeking was 0.80.

**Conclusions:**

The evaluation of the questionnaire for identifying the individuals in the risk of addiction showed that the questionnaire benefits from appropriate validity and reliability. Therefore, it can be used in preventive fields and research. Moreover, by illuminating interpersonal factors that are effective in drug abuse, proper strategies can be used to prevent drug addiction.

## 1. Background

Drug addiction is a chronic brain disease which has deep social, psychological, physical and economic effects. Opium abuse is increasing especially among the youths (1). In Iran, opium abuse is also considered the most important and the biggest concern. Drug-abuse, especially heroine injection, is a serious problem and is considered the most important factor for HIV in the country (2). According to United Nations in 2005, Iran has the highest proportion of the addicted to heroin and opium in the world. Based on this report, of every 17 people in Iran, one person in addicted to these drugs. Moreover, 20 percent of 15 - 60 years old population of Iran is in a way involved in drug abuse. In spite of the fact that there is no exact statistics for the number of drug addicted in the country, UN has declared that there are 10 million addicted in Iran (3). Drug-abuse has caused dramatic social, economic, political and hygienic damages to the country, among which physical and contagious disease such as AIDS, Hepatitis and mental-social disease such as the increase of the crimes connected to addiction, robbery, murder, self-burning, un-employing , aggression, child abuse, the increase in the number of divorces and finally educational problems in children whose parents are addicted can be mentioned (4).

Considering the high costs of the campaign against drugs and treatment of drug-addicted and drawbacks of the existing treatment methods, it seems that preventing drug-abuse is more economic and fruitful. Therefore, the existence of tools for measuring addiction in the country and recognizing the people at risk of drug-abuse can be a very important step in the prevention of drug-abuse. In all countries, it is very difficult to evaluate the amount of consumed drugs. In addition, dependence of the individual to drug is only known when he is completely involved in drug-abuse and is addicted to it. Today one of the common ways of drug abuse recognition is medical test which has two drawbacks, one is that they are costly and the other is that they just show if a person is addicted or not and therefore they are not good for recognizing the people at risk of addiction.

There are various conditions contributing to a tendency to drug-abuse, the most important condition is psycho cognitive traits of the individual, economic, social, cultural and even biological conditions of individuals form special psycho cognitive and behavioral characteristics in the person and determine a special life style for him. The studies done in this field indicate that the lower the level of psychological health of an individual is, the higher is the tendency to drug abuse (5). Many studies are done to know the factors contributing to addiction all of which emphasize on the fact that some temporal, emotional and behavioral disorders play an important role in drug abuse (6). The studies have indicated that 40% of those who are addicted to drugs (opium or non-opium drugs), have had diagnostic criteria of basic depression at a time in their lives (7). The depressed temper and the lack of interest and joy are key signs of depression. A depressed person may express the feelings of sadness, worthlessness and aimlessness (8). Johnson *et al.* (9) state that there is a meaningful correlation between basic depression and addiction to crack. Stress which is severe and unreasonable worry for everything and causes many problems in daily functioning of an individual is considered a psycho-cognitive problem and is found in many drug-addicted. Sometimes an individual tends to use drug and alcohol to control his anxiety and stress. In their study, Liang *et al.* (10) have mentioned depression and stress as predicting factors for addiction and drug abuse. Other recent research studies also support the predicting factor of stress and depression in addiction (11, 12). It is not only temporal disorders which have the road to addiction, sometimes a severe tendency to experience in the new sensations can cause a tendency to drug and finally lead to addiction (13). The studies done on addiction show that the parameter of high sensation-seeking which shows itself in having a tendency to explore sensation, various experiences and taking physical, social, legal and financial risks is significantly correlated which primitive use of cigarette and then continual use of cigarette, drug and alcohol abuse (14). One of the reasons that a sensation-seeking lead to tendency to use drugs is that the level of mono-amin Oxidaz in their brain is low. Therefore they experience joys more deeply than those people who have higher level of this enzyme (13). Furthermore, since adolescence is a time for experiencing, making personal decisions and forming personal identity, teenagers and young people are very vulnerable and at risk of addiction and other dangerous behavior (15). Also the lack of a negative attitude towards drugs and belittling its harmful consequences which is referred to as positive attitude toward drugs is considered a contributing factor to addiction (16).

Therefore considering the growing use of drugs and increasing tendency of Iranians to drug-abuse, the existence of measuring tools to recognize the individuals at risk before they’re addicted is very essential. There is no such instrument in Iran but there are some instruments made in U.S.A or many European countries that are translated to Farsi and adapted to the situation of the country. In Iran Minnesota Multi-phasic Personality Inventory (MMPI-2), MAC-Andrew Alcoholism Scale-R, Addiction Potential Scale (APS) and Addiction Acknowledgment Scale (AAS) are the most frequently-used instruments. Rostami *et al.* (17) investigated these instruments in a study. The result showed that AAS benefits from a good level of precision, MAC-R had a medium precision for distinguishing the people with mental disorders from addicted people, while APS is standardized and localized by Zargar (18) which is named IAPS (Iranian Addiction Potential Scale), and has the lowest exactness and therefore is not considered a proper instrument for diagnosing addiction. Dehkordian (19) conducted a research to recognize the ones at risk of drug-abuse in Iran by making a test for recognizing the individuals in risk of addiction in the students of state universities in Tehran. In this study a 60-questions test was prepared by the researcher and administered. The test could significantly distinguish between addicted and non-addicted individuals. Ekhtiari *et al.* (20) evaluated the reliability and validity of the Persian version of four main questionnaires of Aisenck’s impulsivity, Zackerman’s sensation-seeking, Barratt’s impulsivity and Dickman’s impulsivity through administrating them for healthy and addicted people. The results indicated that Persian version of four Barratt’s questionnaires have proper validity and reliability for determining the level of impulsivity.

Today it's agreed by every professional in the field of instrumentalism making that the content of the instrument should directly be extracted by the people who are the referents of the instrument. The wording and literature of the questions should be taken into account. If the content of any questionnaire is taken by the mental viewpoint of the participants, one can make sure that the instrument can cover all different aspects of the issue under study. In addition, the content of an instrument should also be culturally appropriate. An instrument designed in one special country can only reflect the language and culture of that country and if it is used in another country, many problems arise due to the lack of content-similarity. In this case, even a very careful and good translation of the questionnaire cannot help (21). Since the issue of drug-addiction is an important problem is each country and has cultural, social and economic roots and also considering the fact that there are few valid instruments for addiction in Iran, the present research aims at designing a strong, culturally acceptable instrument for the phenomenon of addiction and the consequent problems in opium-users and then the validity and reliability of the instrument is evaluated and compared to other instruments.

## 2. Objectives

The study was performed to construct the scale for detecting individuals in risk of addiction.

## 3. Materials and Methods

The method of this research is correlation analysis. The aim of a correlational research is to study the range of the differences of one or some variables with the range of differences of one or some other variables. In other words, in the present research the correlation between the test under study and another valid test has been studied. The population in this research consisted young addicted and non-addicted people (between 18 to 35 years old) who live in Tehran.

Sampling conducted randomly through the number of high school and medical science university students in Tehran. The sample were at least 20 individual groups. Also in order to determine the differential validity, addicted population were selected from those young people who had referred voluntarily to the numbers of clinics to give up addiction. Since the frequency and average of the youths at risk of addiction were not accessible, the formula was not used to calculate sample size, but due to the fact that factor analysis is based on the correlation between variables and in sample with small size correlation coefficient doesn’t have the necessary reliability, minimum sample size is critically important for factor analysis. Experts paid attention to various criteria to determine sample size in factor analysis including the minimum proportion of sample to variables. This proportion is mentioned differently in different studies regarding from two samples to one variable to 10 samples to one variable. The experts consider a minimum 200 people sample necessary for the reliability and validity of factor-analysis. According to Comery (22) a range of at least 50 samples in very poor situation to 1000 samples in excellent situation is necessary. He has suggested a guide for determining the sufficient number of sample as follows: 100 samples are very poor, 200 samples are fair, 300 samples are good, 500 are very good and finally a sample more than 1000 or more is excellent. In the present study the primitive form of the questionnaire was administered on 72 non-addicted people and final form was administered on 82 addicted and 150 non-addicted samples. Finally in the present research, 305 people were studied in the initial stage; re-test and final stage in order to achieve the purpose of the study.

To design the researcher-made questionnaire in the field of addiction, first different theories and point of views on addiction and drug-abuse were studied and the views were compared. In addition, some key professionals in the field including psychologists and health and hygiene consultants were interviewed to get more information about addiction and internal factors affection it. The process of sample selection continued until no new information was found.Then, concerning inter-personal factors for addiction, parameters of depression, stress and anxiety, having a positive attitude to drug-abuse, seeking high levels of excitement and self-esteem were selected. Then the information gathered was formed in to questions. In this stage, almost more than 120 questions were prepared while the local culture was considered in the content and form of the questions; therefore those questions that were not suitable for our culture have been replaced with cultural ones. To evaluate the content validity of the questionnaire, 15 academic members of universities and experienced and knowledgeable experts in the field of addiction and questionnaire-making were consulted, so that based on Valtz and Basel content validity index, the level of relativity, clarity and simplicity of the questions could be found out on Likert four degree spectrum (23). For this scale first, relativity of all the questions is measured. If the obtained index equals 79% or higher, that question is an acceptable (24, 25). Compared to other ways, this index benefits from higher objectivity to determine validity (23). To judge face validity of the test, the same professionals who were consulted for content validity and 10 young people were used to judge if the sentences are fluent easily understood and clear. After content validity of the researcher made questionnaire was approved, it was applied on 73 individuals between 18 to 35 years old in April 2012 and then the answers were statistically analyzed in order to change, remove or modify any questions which had a problem or were vague. In this stage five questions were also added to the questionnaire to detect lies. Then the modified form of the questionnaire was again given to professionals and after making suggested changes, the final form of the questionnaire was prepared in May 2012 and along with DASS-21 scale and Zackerman excitement-seeking was applied on 150 non-addicted and 82 addicted young individuals to evaluate its cuncurrent validity. DASS scale for anxiety, which is made by Luice Bands 1995, is a self-assessment 21 part collection which is designed for measuring negative emotional feelings of depression, anxiety and dependence. Its concurrent validity with Back test of stress and anxiety is respectively 81% and 74% (26). In Sahebi, *et al.*'s research (27) the internal correlation (Type Cronbach Alpha) of the questionnaire for depression and anxiety is respectively 77% and 79%. For the scale of having a positive attitude to drug, A.P.S questionnaire was used. The scale which is made by Yadollah Zargar from Shahid Chamran University consists of 34 statements and measures one's tendency to addiction. The scale is modified based on the characteristies of Iranian culture and the validity and reliability of it is approved by experts in the field (27).

Zakerman scale of excitement-seeking which was prepared in 1978 was used to evaluate the concurrent validity of the questionnaire concerning high excitement seeking. The scale has 40 two-part items and measures the factors of excitement-seeking. According to what Zakerman *et al*. (28) has calculated acceptable internal validity ranges between 83% to 86%. After applying the questionnaire and scoring it and in order to determine construct validity of the questionnaire, exploratory factor analyses through "main parameters" with varimax rotation. Which studies internal relationship between variables was used to explore the variables being the most related. In the method of factor analyses, correlated variables are summarized in the form of a new variable called factor. After removing improper questions factor analyses was again run, finally a scale with 75 questions was prepared.

To determine the reliability of the questionnaire for identifying the youths in the risk of addiction, internal correlation method (Cronbach Alpha) was used. Reliability is defined as the existence of correlation and the consistency in the measures of the attributes or constructs (29). Cronbach Alpha represents the degree of coordination in a group of statements making a construct. To have good and sufficient internal correlation, Cronbach Alpha should be between %70-%80 (30).

After administering the questionnaire and its numerating, in order to investigate the factor structure, exploratory factor analysis through basic principles with Varimax rotation had been used. In the factor analysis method, equilibrated variables summerised as new variable form named factor. After deletion of inappropriate questions, once more factor analysis administered. Also to evaluate construct validity, the correlation obtained factors in questionnaire and its subscales with the construct of DASS-21 questionnaire, Zanda’s self-esteem questionnaire and Zakerman sensation-seeking questionnaire was calculated and equaled 0.63 which is meaningful in a level of less than 0.01. At the end the 75-questions scale had been prepared.

## 4. Results

To judge concurrent validity of the questionnaire for identifying the individuals in risk of addiction, the correlation of this instrument with DASS test, I.A.P.S and Zakerman's excitement seeking test was measured, the results of which are in Table 1. The investigation of the results of concurrent validity showed that the questionnaire for identifying the individuals in the risk of addiction has positive and significant correlation with DASS scale, I.H.P.S and Zakerman's excitement seeking test (P ≤ 0.001).

**Table 1.** Results of Correlation Between Identifying People in Risk of Addiction Questionnaire With Equivalent Scales in Order to Evaluating Criterion (Concurrent) Validity

Criterion Tests Questionnaire Subscales	(DASS) Depression Test	(DASS) Anxiety Test	Iranian Addiction Potential Scale (Iaps)	ZackermanHigh Sensation Seeking Test
**Depression**	0.823**	0.678**	0.641**	-0.046**
**Anxiety**	0.575**	0.659**	0.571**	0.53*
**Positive attitude to drug**	0.678**	0.726**	0.737**	-0.044
**High sensation seeking**	0.156**	0.358**	0.247**	0.169*
**Total score of questionnaire**	0.764**	0.722**	0.710**	0.78**

Table 2 shows the mean and standard deviation of the questionnaire for identifying the youths in the risk of addiction and the factors contributing to it. As it is seen, the highest mean is for the whole questionnaire and the factor of depression, while the lowest mean is for having a positive attitude to drug. For evaluating construct validity of the questionnaire, exploratory analyses were used which is shown in Table 3. According to Table 3, KMO test indicates that sample size is appropriate for factor analysis. As it can be seem in Table 4. through the method of exploratory factor analysis, four factors were extracted from the questionnaire after obliging rotation. These four factors are depression with 29 questions, anxiety and stress with 17 questions, having a positive attitude to drug with 18 questions and finally seeking high excitement with 11 questions. Finally after initial evaluation of the questions (for their understandability and face validity) and factor analysis, 75 questions remained to test the risk of tendency to drug. After preparing the 75 questions and the concurrent, face and construct validity of the questionnaire was evaluated, it was given to one addicted and one non-addicted groups to filled in, (Table 5).

**Table 2.** Mean and Standard Deviation Scores of Addicted and non-addicted People in Identifying People in Risk of Addiction Questionnaire and Its Components

	Mean ± SD	t-test	df	Significance
**Depression**		8.450	303	0.001**
Non addicted	20.25 ± 14.149			
Addicted	36.48 ± 16.675			
**Positive attitude to drug**		13.051	303	0.001**
Non addicted	9.27 ± 9.361			
Addicted	25.35 ± 10.015			
**Anxiety**		8.038	303	0.001**
Non addicted	14.75 ± 8.723			
Addicted	23.89 ± 9.013			
**High sensation seeking**		0.972	303	0.332
Non addicted	13.08 ± 5.479			
Addicted	12.32 ± 7.377			
**Total score of questionnaire**		9.958	303	0.001**
Non addicted	57.35 ± 31.883			
Addicted	98.04 ± 30.939			

**Table 3.** KMO & BTS Test for Administration of Sample Size Appropriation[Table-fn fn3547]

Test	Result
**KMO Test**	0.901
**BTS Test**	160570.56
**Df**	136
**Significance**	0.001

Abbreviations: KMO; Test; Kaiser-Meyer-Olkin Test, BTS Test; Bartlett's Test of Sphericity

**Table 4.** Question Factorial of the Identifying People in Risk of Addiction Questionnaire

Factors	Special Value	Justified Variance Percentage
**Depression**	23.221	5.215
**Anxiety**	6.682	11.888
**Positive attitude to drug**	5.886	14.143
**High sensation seeking**	2.250	5.473

**Table 5.** The Questions and the Percentages of Their Contribution to Drug-Addiction in the Questionnaire for Identifying Individuals in Risk

No.	Statement	Percentage of Contribution to Drug Addiction (%)
**1**	I feel something bad is going to happen to me.	47
**2**	Nothing can make me happy.	66
**3**	I feel I don't deserve the good things in my life.	56
**4**	I like taking part in exciting and noisy parties.	61
**5**	Drug-abuse is not a bad way to get rid of the problems of life.	78
**6**	I feel worried, stressed and anxious.	56
**7**	There are very few things to make me happy.	62
**8**	I feel upset that I don't have what I wished.	54
**9**	I believe life is worthless and I should have fun anyway.	55
**10**	When I'm very sad I like to make calm by drug.	67
**11**	I feel horrified and have fast heart beat without and reasons.	58
**12**	I'm nagging and pessimistic.	42
**13**	I care about what people think of me.	46
**14**	The only thing that matters to me is having fun. I don't care about other people's ideas.	49
**15**	Drug-abuse is very common in our neighborhood.	45
**16**	I have nightmares of jump a wake in the middle of the night.	42
**17**	I think people would be happier if I was dead.	70
**18**	I've missed many opportunities since I can't decide myself.	58
**19**	To escape routine life, I need something beyond the existing excitement.	55
**20**	I think I can experience very interesting feelings by drug-abuse.	72
**21**	I don't think I can make success in my career any time.	58
**22**	I feel lonely and not loved by anyone.	64
**23**	I feel people don't pay me any respect.	62
**24**	I like trying new excitement.	58
**25**	Drug-abuse is something common among my friends.	71
**26**	I spend a lot of time worrying about the future.	59
**27**	Recently I hate myself.	76
**28**	If I don't like a behavior of one of my friends or relatives, I can't tell them.	51
**29**	How much I enjoy a party depends on its fun.	62
**30**	I believe in order to escape the problems of life; drug is a much easier way compared to other ways.	76
**31**	I feel terrified when I think I'm completely alone in this world.	50
**32**	I've lost interested in everybody and everything.	67
**33**	I think people don't like making friends with me.	66
**34**	I've already had minor sexual problems and deviations.	35
**35**	Drug-abuse for fun doesn't make anybody addicted.	58
**36**	I suffer from a mental or physical pain.	57
**37**	I think others are happier them me.	56
**38**	Talking in front of others is hard for me.	50
**39**	To enjoy the group, you should follow whatever friends say.	39
**40**	Occasionally, my friends have offered me drug.	
**41**	I don't feel relaxed and cannot keep relaxed easily.	60
**42**	I don't think I'm as happy as others.	52
**43**	My friends don't trust me.	39
**44**	I like gambling even if I don't have financially good situation.	39
**45**	Drug can increase awareness, concentration and strength.	68
**46**	I feel stressed.	69
**47**	I feel lonely.	60
**48**	I feel worried that others may consider me unsuccessful and failed.	48
**49**	My behavior is unpredictable and unexpected for others.	50
**50**	To remove some physical pains it's not bad to use drug.	71
**51**	Sometimes I feel too upset to stay still.	60
**52**	I have lost my hope in future.	61
**53**	I suffer from inferiority complex.	52
**54**	In parties and gatherings I tend to amplify my happiness by using drugs that are less addictive.	71
**55**	I don't think communicating with addicted people is wrong.	59
**56**	My body shakes with fear without any logical reason.	49
**57**	Even accomplishing simple and small tasks have become difficult for me.	50
**58**	I don't feel satisfied with my present situation.	40
**59**	I'm easily excited.	36
**60**	I've used alcoholic drinks or drug at least once.	50
**61**	I stammer in situations that I feel social pressure.	53
**62**	I feel confused in life.	56
**63**	I feel I'm not loved by other.	61
**64**	I like to feel good through stimulating drug.	65
**65**	Drug-abuse is not as problematic as they say. It depends on the individual.	66
**66**	For no good reason I feel stressed and I have butterflies in my stomach.	49
**67**	I'm slow in doing my personal affairs.	49
**68**	Over all, I don't have a positive attitude toward myself.	60
**69**	I like doing activities that are highly exciting.	66
**70**	I think for the person who has many problems, drug is a good way to forget about the problems.	72
**71**	I'm extremely worried about the future	55
**72**	I feel failed in the majority of cases.	58
**73**	It's hard for me to encounter my problems and I avoid facing them.	42
**74**	I'm continuously seeking other people's attention.	40
**75**	Drug seems a good way to get rid of boredom.	79

As it is shown in Table 2, the addicted and non-addicted groups are significantly different regarding the psychological parameters. The means of the addicted group in the parameters of depression, anxiety, having a positive attitude to drug, and high excitement seeking are significantly higher than those of the non-addicted group (P ≤ 0.001). The biggest difference is related to the factor of depression, while the smallest one is related to having a positive attitude to drug.

Total reliability coefficient of the questionnaire for identifying the individuals in the risk of addiction along with reliability coefficients of its four subscales are shown in Table 6. All the questions had positive correlation with the total scores of each factor. As it's seen in Table 6, total reliability coefficient of the questionnaire (Type Cronbach Alpha) and reliability coefficients of its subscales are all bigger than 0.7 which reveal the acceptable reliability of the questionnaire.

**Table 6.** Validity Coefficients (Internal Similitude) Of Identifying People in Risk Of Addiction

	Number of Questions	Internal Similitude Coefficient	Guttmann Coefficient
**Depression**	29	0.96	0.94
**Positive attitude to drug**	18	0.93	0.91
**Anxiety**	17	0.90	0.88
**High sensation seeking**	11	0.80	0.78
**Total score of questionnaire**	75	0.97	0.95

## 5. Discussion

Considering the importance of the problem of addiction and the increasing tendency of the youths toward drug abuse, the present research has been conducted to pave the ground for identifying some inter-personal and psychological factors contributing to addiction and to encourage basic and practical further research. High and significant correlation at this questionnaire with the questionnaire by Dos, I.A.P.S and excitement-seeking scale of Zackerman prove acceptable concurrent validity of the questionnaire. The result of factor analyses revealed the appropriate content validity of the test and showed that 46.71% of the reasons for having a tendency to drug-abuse and addiction can be explained by four factors of depression, anxiety, having a positive attitude to drug and seeking excitement. The questions in the questionnaire were approved by a professional team in the field of study; therefore it benefits from acceptable face and content validity. In addition, to make sure of the validity of the questionnaire for identifying the individuals in risk, the test was applied on two groups: one addicted and one non-addicted. The results showed the addicted group had significantly higher scores in the parameters of depression, anxiety, having a positive attitude to drug and seeking excitement. Considering this finding, it can be concluded that this instrument benefits from good validity for identification. Internal correlation coefficient (Type Cronbach Alpha) for the whole questionnaire equaled %97 and for the subscales was about 0.80 to 0.96 which indicates high reliability of it.

The findings of the research are along with the findings of Worley *et al.* (31), Greenfield *et al.* (32), Tsai *et al.* (33), Wolitzky *et al.* (34), Zabela *et al.* (35) in which psychological parameters of depression, anxiety, having a positive attitude to drug and excitement-seeking are correlated with drug addiction. In their research, Worley *et.al.* (31) showed many addicted people have personality problems such as high sensitivity, emotional disorders like bad temperedness, depression, abnormal excitement, and temporary forgetfulness. In addition, many researchers believe that many addicted people use drug to reduce their stress and aggressive behavior (32).

Moreover, according to Tsai *et al.* (33), having a positive attitude to drug and the belief that "I don't get addicted", "I'm different", and "Marihuana and grass are not addictive" make the person prepared for drug-abuse (34). People don't get addicted all of a sudden and overnight. They get actively involved in drug and here their beliefs, purposes, attitudes and expectations play an important role. In a research by Zabela *et al.* (35) it was found out that seeking high excitement has an intervening role in physiological stimulations related to risk-taking and has a meaningful correlation with drug-abuse. Among the limitations of the present research, limited age range of the participants, lack of test-retest reliability and the application of the instrument just for literate people can be mentioned. It's suggested that factor-analyses be done on a bigger sample if possible. Since the addicted people participating in this study were volunteers to give up drug-abuse in clinics, it's recommended that the instrument be applied on those addicted people who don't decide to give up too.
